# Musculoskeletal Disorders Associated With Quality of Life and Body Composition in Urban and Rural Public School Teachers

**DOI:** 10.3389/fpubh.2021.607318

**Published:** 2021-06-01

**Authors:** Gustavo Vega-Fernández, Lydia Lera, Bárbara Leyton, Pilar Cortés, Pablo A. Lizana

**Affiliations:** ^1^Laboratory of Morphological Sciences, Instituto de Biología, Pontificia Universidad Católica de Valparaíso, Valparaíso, Chile; ^2^Programa de Magister en Ciencias Mención Morfología, Universidad de La Frontera, Temuco, Chile; ^3^Public Nutrition Unit, Institute of Nutrition and Food Technology, University of Chile, Santiago, Chile

**Keywords:** mental health, physical health, quality of life, school teachers, obesity, musculoskeletal disorders

## Abstract

**Introduction:** Teachers have been reported to be a labor group with high rates of musculoskeletal disorders (MSDs), stress, and strong deterioration of quality of life (QoL). However, little information exists about the association between MSD, QoL, and body composition in rural and urban teachers.

**Objective:** The aim was to study the association of MSD with QoL perception and body composition of urban and rural teachers.

**Participants and Methods:** Participants are comprised a representative sample of urban and rural public schoolteachers from the Valparaiso Region, Chile. MSDs were evaluated with the Standardized Nordic Questionnaire for Musculoskeletal Symptoms validated for the Chilean population. QoL perception was evaluated with the 36-Item Short-Form Survey (SF-36). Body composition was measured via bioimpedance. A logistic regression model was used to evaluate the association between MSD, QoL, and body composition, adjusted for age and gender.

**Results:** A total of 88.9% (urban 90%; rural 87%) of teachers felt pain in some body area, 71.2% of them with limitations; 39% of teachers presented body fat obesity, with the highest rate in rural women. The body area with the greatest MSD prevalence was the neck and shoulders (68.6%). Significant differences were observed between teachers with >p75 of MSD (over six pain regions) and those with ≤p75 (six or fewer painful regions; *p* < 0.05) on six QoL scales and on physical health components (PCSs) and mental health (MCS) in urban teachers. However, rural teachers presented no differences. The association between teachers with >p75 MSD and low QoL perception was significant (*p* < 0.05) in PCS and MCS. Furthermore, the regression model presents a significant association between rural areas and low PCS perception.

**Conclusions:** Urban and rural teachers present high rates of MSD and obesity. Teachers with higher rates of MSD have their mental and physical QoL affected, making workplace intervention in MSD necessary to prevent teacher health deterioration.

## Introduction

Musculoskeletal disorders (MSDs) are pain symptomatologies caused by damage to the locomotive system, produced by external action, high biomechanical exposure, or psychosocial or labor-psychological factors (1–4). MSDs can present as acute or chronic problems and can be incapacitating for their sufferers (5, 6), leading to high costs for health systems (7), especially for chronic pain (8).

MSDs are associated with jobs where people are exposed to work overload, both physical and mental (3). One of the professions with the highest MSD rate worldwide is school teaching (9–11). Furthermore, a close relation exists between MSD and classroom experience years, age range, female gender (12), obesity (13–15), school type and number of students assigned (16), school infrastructure, work conditions, high stress, and psychosocial factors (4, 10, 17–19), with links even being observed with the urban or rural area where teachers work (20). MSDs have been studied in both developed and developing countries, with high prevalence in body segments including the lower back, neck, shoulders, and upper limbs (14, 21–23). In this way, evidence exists regarding the relation between MSD and quality of life (QoL) perception in teachers (10, 11, 24). Among the Chilean schoolteacher population, physical and mental risk factors have already been reported (25, 26) to be related to both QoL and body composition (27), suggesting that Chilean schoolteachers could be at risk for MSD, which has no extant evidence in the literature. Therefore, the objective of this study is to evaluate MSD prevalence in Chilean urban and rural teachers and its association with QoL and body composition.

## Materials and Methods

### Participants

Chilean school administration includes public schools, subsidized charter schools (copay), and private schools, which are located in rural and urban sectors. According to Chilean Education Ministry data, Chilean teachers are 88.1% urban and 11.9% rural (28).

This cross-sectional study was conducted among public schools because rural schools are public, allowing comparison between the same type of administration. Fourteen rural schools and six urban schools were randomly selected. All teachers working in those schools were invited to participate in the study. The initial sample consisted of a total of 218 teachers who agreed to take part in the study, corresponding to the cities of Hijuelas, LlayLlay, La Calera, Valparaíso, and Viña del Mar. Sample size was calculated based on MSD data from the Chilean working population (29), which reported a prevalence of 49.8%, considering a precision of 10%, a potency of 80%, and an alpha of 5%, obtaining a total sample of 149 participants (urban and rural). The initial sample was overestimated by possible losses. Finally, 65 subjects were excluded for the following reasons: Nordic MSD questionnaire non-completion (40), not presenting the body composition evaluation (14), and incomplete 36-Item Short-Form Survey (SF-36) QoL instrument data (11). Therefore, the final sample consisted of 153 teachers.

### Instruments

#### Anthropometry and Body Composition

To calculate body mass index (BMI: kilograms/size in square meters), nutritional status categories for BMI were determined using WHO criteria (30): underweight (BMI < 18.5 kg/m^2^), normal weight (18.5 kg/m^2^ ≤ BMI < 25 kg/m^2^), overweight (25 kg/m^2^ ≤ BMI < 30 kg/m^2^), or obese (BMI ≥ 30 kg/m^2^). Size was measured using a mobile SECA 213 stadiometer. For body composition evaluation, a bioelectrical impedance device was used (TANITA BC 240 SMA, Tanita, Tokyo, Japan). Before evaluation, participants were asked to not consume alcohol in the previous 48 h, not to carry metal objects, not to consume any caffeine or diuretics in the previous 4 h, not to have done intense exercise in the previous 12 h, and to urinate within 30 min before the test. Body composition variables considered in this study were as follows: fatty mass (FM; in kg and %), fat-free mass (FFM; in kg and %), and muscle mass (MM; in kg and %). To classify obesity, %FM was used with the following cutoff points: >35% for women and >25 for men (31).

### Quality of Life

QoL perception was evaluated with the SF-36, validated for the Chilean population (32). The questionnaire consists of 36 Likert-type personal appreciation questions grouped into eight scales: physical function (PF), physical role (PR), body pain (BP), general health (GH), vitality (VT), social function (SF), emotional role (RE), and mental health (MH). Furthermore, these scales are grouped into two categories: physical health component (PCS) and mental health component (MCS). Participants' scores for each scale and component were transformed into a 0–100 scale, after which a z-score and a t-score were calculated for each scale and measurement PCS and MCS with the internationally standardized method (33). Finally, the worst scores were grouped in the ≤50th percentile for PCS and MCS.

### Musculoskeletal Disorders

For MSD evaluation, the Standardized Nordic Questionnaire for Musculoskeletal Symptoms was used, validated for the Chilean population (34). This instrument grants information about teachers' pain prevalence in 12 body areas: neck, right shoulder, left shoulder, right forearm/elbow, left forearm/elbow, right hand/wrist, left hand/wrist, upper back, lower back, hips/buttocks/thighs, knees, and feet/ankles. The first part of the questionnaire consisted in detecting whether or not pain was present in any of the 12 previously mentioned areas during the last 12 months. The second part of the questionnaire investigated whether incapacitating pain happened within any of the 12 body areas in the last 12 months.

### Proceedings

Participating teachers signed voluntary informed consent prior to background collection, explicitly stating that all personal results are strictly confidential. All proceedings for this study were approved by the bioethics committee at Pontificia Universidad Católica de Valparaíso.

### Statistical Analysis

Data analysis was done with STATA MP2 V.16 software for Windows. Statistical description was done using mean with standard deviation (M ± SD) for continuous variables and frequencies with percentages for categorical variables (*n*, %). Comparisons of sociodemographic, anthropometric, and body composition variables in urban and rural teachers were made between genders. MSD rates were presented as frequency and percentage (*n*, %) according to presence in the previous 12 months (12M) or limited presence in the previous 12 months (12M-Lim) in urban and rural teachers. Subjects with higher amounts of painful regions were evaluated by grouping them in the 50th and 75th percentiles, according to the previous 12 months (p50 > 4 regions, p75 > 6 regions) and the previous 12 months with limitations (p50 > 2 regions, p75 > 5 regions). Sociodemographic, anthropometric, body composition, and MSD characteristics were compared between those teachers with high and low scores (t-scores) for each scale of QoL, and 50th percentile in the QoL PCS and MCS (≤ p50 and > p50 respectively). Thus, scores above 50 indicate a better QoL and scores below 50 indicate a worse QoL. QoL was also compared on each scale, among those teachers with more than six painful areas (>p75) and with six or fewer painful areas (≤p75) in the urban and rural groups. Comparisons were used according to data distribution in accordance with the Shapiro–Wilk normality test (*t*-test: parametric; Mann–Whitney: nonparametric). The chi-squared and Fisher exact association tests were used to analyze categorical variables. Logistical regression was also done to analyze association between the most prevalent MSD cases (>p75), obesity, and urban–rural area with QoL in its PCS and MCS measurements, adjusted for gender and age.

## Results

### Sociodemographic Characteristics

Out of 153 participants, 71.2% were women with ages between 23 and 68 years (median ± standard deviation: 38.65 ± 11.65). Urban teachers represented 53.6%, and rural teachers were 46.4% of the total sample. In Table 1, sociodemographic, body composition, and anthropometric measurement characteristics can be observed grouped by total sample and geographic area and analyzed between genders. Women teaching in rural establishments presented significantly more housework hours than men (*p* < 0.05). No significant differences exist between genders in any of the marital status, contract type, and domestic work variables.

**Table 1:** Sociodemographic and body composition evaluated by gender in urban and rural teachers in the Valparaíso region, Chile.

	**Total sample**			**Urban (*****n*** **=** **82)**			**Rural (*****n*** **=** **71)**		
	**Male**	**Female**	***p***	**Male (*n* = 25)**	**Female (*n* = 57)**	***p***	**Male (*n* = 19)**	**Female (*n* = 52)**	***p***
Age (years)^a^	39.95 ± 14.28	38.12 ± 10.44	0.963^d^	37.24 ± 13.33	36.49 ± 9.61	0.586^d^	49.52 ± 15.05	39.90 ± 11.10	0.487^d^
≤44^b^	29 (65.9)	79 (72.5)	0.420^f^	19 (76.0)	46 (80.7)	0.629^f^	10 (52.6)	33 (62.3)	0.408^f^
≥45^b^	15 (34.1)	30 (27.5)		6 (24.0)	11 (19.3)		9 (47.4)	19 (35.5)	
Marital status									
Single^b^	20 (45.5)	51 (47.2)	0.183^g^	15 (60.0)	31 (54.4)	0.131^g^	5 (26.3)	20 (39.2)	0.603f^g^
Couple^b^	8 (18.2)	9 (8.3)		5 (20.0)	5 (8.8)		3 (15.8)	4 (7.8)	
Married^b^	14 (31.8)	35 (32.4)		5 (20.0)	13 (22.8)		9 (47.4)	22 (43.1)	
DWW^b^	2 (4.5)	13 (12.0)		0 (0)	8 (14.0)		2 (10.5)	5 (9.8)	
Type of contract									
Fixed-term^b^	24 (58.5)	48(44.9)	0.136^f^	16 (69.6)	36 (64.3)	0.653^f^	8 (44.4)	12 (23.5)	0.093^f^
Indefinite-term^b^	17 (41.5)	59 (55.1)		7 (30.4)	20 (35.7)		10 (55.6)	39 (76,5)	
Domestic work									
<15^b^	41 (93.2)	91 (85.0)	0.279^g^	22 (88.0)	50 (89.3)	0.999^g^	19 (100)	41 (80.4)	0.037^g^
≥15^b^	3 (6.8)	16 (15.0)		3 (12.0)	6 (10.7)		0 (0.0)	10 (19.6)	
Height^a^	173.05 ± 6.12	158.96 ± 5.92	<0.001^e^	175.32 ± 5.77	159.70 ± 6.06	<0.001^e^	170.05 ± 5.33	158.15 ± 5.71	<0.001^e^
Weight^a^	79.361 ± 10.91	68.190 ± 13.36	<0.001^d^	80.804 ± 11.64	66.451 ± 13.07	<0.001^e^	77.463 ± 9.86	70.096 ± 13.55	<0.008^c^
BMI (kg/m2)^a^	26.523 ± 3.60	26.942 ± 4.55	0.618^d^	26.288 ± 3.65	26.205 ± 4.16	0.932^e^	26.832 ± 3.62	27.750 ± 4.85	0.475^d^
Eutropic^b^	16 (36.4)	41 (37.6)	0.309^f^	10 (40.0)	25 (43.9)	0.945^g^	6 (31.6)	16 (30.8)	0.262^g^
Overweight^b^	23 (52.3)	45 (41.3)		12 (48.0)	24 (42.1)		11 (57.9)	21 (40.4)	
Obese^b^	5 (11.4)	23 (21.1)		3 (12.0)	8 (14.0)		2 (10.5)	15 (28.8)	
FM (kg)^a^, ^c^	18.839 ± 7.47	23.809 ± 9.46	0.001^d^	17.420 ± 6.95	21.519 ± 8.39	0.035^e^	20.705 ± 7.89	26.319 ± 9.99	0.022^d^
FM (%)^a^, ^c^	23.155 ± 6.90	33.570 ± 7.48	<0.001^e^	20.900 ± 5.837	31.011 ± 7.02	<0.001^e^	26.121 ± 7.21	36.375 ± 7.02	<0.001^e^
Normal^b^	30 (68.2)	63 (57.8)	0.234^f^	19 (76.0)	40 (70.2)	0.589^f^	11 (57.9)	23 (42.2)	0.308^f^
Obese^b^	14 (31.8)	46 (42.2)		6 (24.0)	17 (29.8)		8 (42.1)	29 (55.8)	
FFM (kg)^a^, ^c^	60.522 ± 6.43	44.700 ± 4.72	<0.001^d^	63.348 ± 5.89	45.505 ± 5.409	<0.001^e^	56.757 ± 5.10	43.818 ± 4.21	<0.001^e^
FFM (%)^a^, ^c^	76.167 ± 8.28	66.474 ± 7.42	<0.001^d^	79.104 ± 5.83	68.989 ± 7.02	<0.001^e^	72.303 ± 9.52	63.717 ± 6.91	<0.001^e^
MM (kg)^a^, ^c^	57.470 ± 6.10	42.381 ± 4.47	<0.001^d^	60.220 ± 5.62	43.135 ± 4.79	<0.001^d^	53.853 ± 4.77	41.554 ± 3.97	<0.001^e^
MM (%)^a^, ^c^	72.973 ± 6.56	63.480 ± 8.41	<0.001^d^	75.152 ± 5.53	66.280 ± 8.94	<0.001^d^	70.105 ± 6.85	60.410 ± 6.61	<0.001^e^

*BMI, body mass index; ≤44 to ≥45, age categories (years); DWW, divorced, widow, widower; BMI, body mass index; FM, fat mass (kg and %); FFM, free-fat mass (kg and %); MM, muscle mass (kg and %); Domestic work, <15 to >15 (hours per week)*.

a *Mean ± SD, standard deviation*.

b *Data are expressed as frequency (percentage)*.

c *Body components evaluated by bioimpedance (TANITA BC 240 SMA, Tanita, Tokyo, Japan)*.

d *Mann–Whitney test*.

e *t-test*.

f *Chi-squared*.

g *Fisher's exact test. p < 0.05*.

### Body Composition

Between males and females, obesity measured in BMI and %BF presented no significant differences in any of the samples. However, rural women presented the highest obesity rates in both BMI and %BF (28.8 and 55.8%, respectively). Furthermore, the FFM (kg), FFM (%), MM (kg), and MM(%) body composition variables presented significantly low values for females (*p* ≤ 0.001 for all), in urban and rural teachers (Table 1).

### Musculoskeletal Disorders

In Table 2, we can observe that 88.9% of teachers felt pain in at least one body area and 71.2% felt pain with limitations. High MSD rates are observed in both sectors (urban 90.2%; rural, 87.3%). By body area, the greatest pain prevalence in the previous 12 months is in the neck and shoulder regions (68.6%), and 54% of teachers presented limitations in their activities due to neck and shoulder pain in the previous 12 months. In geographical area comparison, neck and shoulder pain is more prevalent in urban teachers (76.8%), and 64.6% presented limitations due to said pain. Additionally, the lower back is another region with high MSD prevalence rates in urban teachers (70.7%), and 56.1% presented limitations. The upper limb was the anatomical segment with the most MSD in 79.7% of teachers.

**Table 2:** MSD prevalence among urban and rural teachers in the Valparaíso region of Chile.

	**Prevalence 12M** ***n*** **(%)**			**Prevalence 12M-Lim** ***n*** **(%)**		
	**Total**	**Urban**	**Rural**	**Total**	**Urban**	**Rural**
Neck	86 (56.2)	53 (64.6)	33 (46.5)	68 (44.4)	45 (54.9)	23 (32.4)
Shoulders	71 (46.4)	42 (51.2)	29 (40.8)	50 (32.7)	34 (41.5)	16 (22.5)
Neck/Shoulders	105 (68.6)	63 (76.8)	42 (59.2)	82 (53.6)	53 (64.6)	29 (40.8)
Elbows	32 (20.9)	20 (24.4)	12 (16.9)	21 (13.7)	13 (15.9)	8 (11.3)
Wrist/Hands	69 (45.1)	36 (43.9)	33 (46.5)	41 (26.8)	26 (31.7)	15 (21.1)
Any upper limb	122 (79.7)	69 (84.1)	53 (74.6)	94 (61.4)	57 (69.5)	37 (52.1)
Upper back	69 (45.1)	41 (50.0)	28 (39.4)	49 (32.0)	31 (37.8)	18 (25.4)
Low back	88 (57.5)	58 (70.7)	30 (42.3)	66 (43.1)	46 (56.1)	20 (28.2)
Any Back	102 (66.7)	62 (75.6)	40 (56.3)	78 (51.0)	51 (62.2	27 (38.0)
Hips/Thighs	44 (28.8)	25 (30.5)	19 (26.8)	37 (24.2)	22 (26.8)	15 (21.1)
Knees	63 (41.2)	34 (41.5)	29 (40.8)	32 (34.0)	27 (32.9)	25 (35.2)
Ankles/Feet	50 (32.7)	31 (37.8)	19 (26.8)	38 (24.8)	24 (29.3)	14 (19.7)
Any lower limb	97 (63.64)	56 (68.3)	41 (57.7)	75 (49.0)	45 (54.9)	30 (42.3)
Any MSD	136 (88.9)	74 (90.2)	62 (87.3)	109 (71.2)	61 (74.4)	48 (67.6)
>p50^a^	66 (43.1)	40 (48.8)	26 (36.6)	75 (49.0)	49 (59.8)	26 (36.6)
>p75^b^	32 (20.9)	21 (25.6)	11 (15.5)	30 (19.6)	20 (24.4)	10 (14.1)

*Data are expressed as frequency (percentage); 12M, pain in the last 12 months; 12M-lim, pain limitation in the last 12 months; MSD, musculoskeletal disorder*.

a *>p50, MSD in more than four regions in 12M and more than two in 12M-lim*.

b *>p75, MSP in more than six regions in 12M and more than five in 12M-lim. p < 0.05*.

Cutoff point p50 and p75 MSD presented 48.8% of urban teachers with over four painful areas (>p50) and 25.6% over six regions (>p75), with rural teachers presenting 36.6 and 15.5%, respectively (see Table 2).

In Table 3, associations can be observed between QoL scores (≤50 and >*p*50) in the PCS and MCS. PCS summary measure presents significant associations for area (*p* < 0.05), with 55.8% (*p* < 0.05) of rural teachers presenting low QoL scores. Furthermore, teachers around p50 MSD (≤4 vs. >4 painful areas) and p75 MSD (≤6 vs. >6 painful areas) presented a significant association with the PCS summary measurement of QoL (*p* < 0.05). The MCS summary measurement for QoL presented a significant association between the age categories (*p* < 0.01), where teachers under 45 years had the lowest score in MCS (81%). Furthermore, those teachers with greater MSD (>p75) have a significant association with the MCS (*p* < 0.05), where 29% of teachers have low scores.

**Table 3:** Sociodemographic characteristics, body composition, and MSD according to QoL PCS and MSC.

**Variable**	**Total sample**	**PCS**		***p***	**MCS**		***p***
		**> 50 percentil**	**≤ 50 percentil**		**> 50 percentil**	**≤ 50 percentil**	
Gender							
Male^b^	44 (28.8)	26 (34.2)	18 (23.3)	0.139^f^	26 (34.2)	18 (23.8)	0.139^f^
Female^b^	109 (71.2)	50 (65.7)	59 (76.6)		50 (65.8)	59 (76.2)	
Area							
Urban^b^	82 (53.6)	48 (63.1)	34 (44.16)	0.018^f^	40 (52.6)	42 (54.5)	0.212^f^
Rural^b^	71 (46.4)	28 (36.8)	43 (55.8)		36 (47.3)	35 (45.4)	
Age (years)^a^	38.65 ± 11.66	38.68 ± 11.15	38.61 ± 12.22	0.488^d^	41.12 ± 12.43	36.21 ± 10.35	0.021^d^
<45^b^	108 (70.6)	57 (75.0)	51 (66.2)	0.234^f^	46 (60.5)	62 (80.5)	0.007^f^
≥45^b^	45 (29.4)	19 (25.0)	26 (33.7)		30 (39.4)	15 (19.4)	
Marital status							
Single^b^	71 (46.7)	37 (49.3)	34 (44.1)	0.747^f^	38 (50.0)	33 (43.4)	0.078^g^
Couple^b^	17 (11.1)	9 (12.0)	8 (10.3)		4 (5.3)	13 (17.1)	
Married^b^	49 (32.2)	21 (28.0)	28 (36.3)		24 (31.5)	25 (32.8)	
DWW^b^	15 (9.8)	8 (10.6)	7 (9.1)		10 (13.1)	5 (6.6)	
Type of contract							
Fixed^b^	72 (48.6)	37 (49.3)	35 (48.0)	0.866^e^	33 (44.5)	39 (52.7)	0.324^e^
Indefinite^b^	76 (51.3)	38 (50.7)	38 (52.0)		41 (55.4)	35 (47.3)	
Domestic work							
<15^b^	132 (87.4)	68 (90.7)	64 (84.2)	0.232^e^	61.(82.4)	71 (92.2)	0.070^f^
>15^b^	19 (12.6)	7 (9.3)	12 (15.7)		13 (17.6)	6 (7.8)	
Height(m)^a^, ^c^	163.01 ± 8.74	164.17 ± 9.22	161.87 ± 8.14	0.197^d^	163.87 ± 9.09	162.17 ± 8.36	0.230^e^
Weight (kg)^a^, ^c^	71.403 ± 13.65	71.669 ± 12.41	71.139 ± 14.85	0.810^e^	73.237 ± 13.20	69.592 ± 13.94	0.047^d^
BMI(kg/m2)^a^, ^c^	26.822 ± 4.30	26.568 ± 3.98	27.071 ± 4.60	0.614^d^	27.261 ± 4.23	26.387 ± 4.35	0.152^d^
Eutrophic^b^	57 (37.3)	30 (39.5)	27 (35.1)	0.662^e^	26 (34.2)	31 (40.3)	0.728^e^
Overweight^b^	68 (44.4)	31 (40.8)	37 (48.1)		35 (46.1)	33 (42.9)	
Obesity^b^	28 (18.3)	15 (19.7)	13 (16.8)		15 (19.7)	13 (16.9)	
FM (kg)^a^, ^c^	22.380 ± 9.19	21.359 ± 8.58	23.387 ± 9.70	0.371^d^	23.302 ± 8.89	21.468 ± 9.44	0.185^d^
FM (%)^a^, ^c^	30.575 ± 8.70	29.289 ± 9.13	31.842 ± 8.12	0.069^e^	31.251 ± 8.45	29.906 ± 8.94	0.340^e^
Normal^b^	93 (60.8)	51 (67.1)	42 (54.6)	0.112^e^	44 (57.9)	49 (63.6)	0.467
Obese^b^	60 (39.2)	25 (32.8)	35 (45.4)		32 (42.1)	28 (36.4)	
FFM (kg)^a^, ^c^	49.250 ± 8.90	50.339 ± 9.25	48.176 ± 8.45	0.100^d^	50.276 ± 9.12	48.238 ± 8.61	0.151^d^
FFM (%)^a^, ^c^	69.261 ± 8.827	70.376 ± 9.42	68.161 ± 8.12	0.121^e^	68.757 ± 8.45	69.760 ± 9.21	0.484^e^
MM (kg)^a^, ^c^	46.720 ± 8.47	47.742 ± 8.79	45.712 ± 8.07	0.096^d^	47.718 ± 8.66	45.735 ± 8.21	0.138^d^
MM (%)^a^, ^c^	66.210 ± 9.00	67.116 ± 8.67	65.316 ± 9.30	0.209^d^	65.87 ± 9.56	66.548 ± 8.47	0.525^d^
p50 MSD							
≤4 regiones^b^	87 (56.9)	53 (69.7)	34 (44.2)	0.001^f^	47 (61.8)	40 (51.9)	0.217^f^
>4 regiones^b^	66 (43.1)	23 (30.1)	43 (55.84)		29 (38.2)	37 (48.1)	
p75 MSD							
≤6 regiones^b^	121 (79.1)	68 (89.47)	53 (68.8)	0.002^f^	66 (86.8)	55 (71.4)	0.019^f^
>6 regiones^b^	32 (20.9)	8 (10.5)	24 (31.2)		10 (13.2)	22 (28.6)	

*QoL, quality of life; PCS, physical component summary; MCS, mental component summary;≤44 to ≥45, age categories (years); DWW, divorced, widow, widower; BMI, body mass index; FM, fat mass (kg and %); FFM, free fat mass (kg and %); MM, muscle mass (kg and %); Domestic Work, <15 to >15 (hours per week); MSD, musculoskeletal disorders, ≤4 to >4 regions (p50), ≤6 to >6 regions (p75)*.

a *Mean ± SD*.

b *Data are expressed as frequency (percentage)*.

c *Body components evaluated by bioimpedance (TANITA BC 420SMA)*.

d *Mann–Whitney test*.

e *t-test*.

f *Chi-squared*.

g *Fisher's exact test. p < 0.05*.

### Quality of Life Perception

Table 4 shows the QoL scores for urban and rural teachers. Differences are observable between subjects with more MSD areas according to the categories ≤p75 vs. >p75. Urban teachers presented significant QoL perception differences on the PF, PR, BP, GH, SF, and RE scales (*p* < 0.05). Both MCS and PCS summary measures were also notably different in urban teachers (*p* < 0.05).

**Table 4:** Comparison between MSD of rural and urban teachers in the eight QoL scales and their summary measures PCS and MCS.

**QoL**	**Total sample**		***p***	**Urban (*****n*** **=** **82)**		***p***	**Rural (*****n*** **=** **71)**		***p***
	**≤p75**	**>p75**		**≤p75**	**>p75**		**≤p75**	**>p75**	
PF	52.889 ± 4.83	51.037 ± 5.99	0.039^a^	53.584 ± 4.69	52.194 ± 3.90	0.039^a^	52.184 ± 4.90	48.829 ± 8.53	0.223^a^
RP	49.694 ± 5.99	47.125 ± 6.79	0.059^a^	50.530 ± 5.68	46.405 ± 7.34	0.027^a^	48.844 ± 6.21	48.502 ± 5.66	0.797^a^
BP	44.032 ± 11.26	38.885 ± 6.77	0.014^b^	46.331 ± 9.84	39.966 ± 5.65	0.006^b^	41.694 ± 12.19	36.821 ± 8.44	0.209^b^
GH	47.305 ± 9.13	41.310 ± 8.06	<0.001^b^	49.471 ± 9.34	41.111 ± 8.45	<0.001^b^	45.102 ± 8.43	41.690 ± 7.64	0.216^b^
VT	47.401 ± 9.16	43.11 ± 7.45	0.015^b^	47.943 ± 9.48	43.705 ± 8.05	0.071^b^	46.849 ± 8.89	41.980 ± 6.35	0.088^b^
SF	43.341 ± 10.12	36.730 ± 9.24	0.003^a^	44.876 ± 9.80	38.177 ± 9.11	0.007^b^	41.781 ± 10.29	33.969 ± 9.29	0.065^a^
RE	48.928 ± 6.53	45.019 ± 7.01	0.005^a^	48.756 ± 6.29	44.249 ± 7.92	0.009^b^	49.103 ± 6.81	46.488 ± 4.82	0.130^a^
MH	44.926 ± 9.823	40.892 ± 9.63	0.039^b^	45.993 ± 11.06	40.966 ± 10.83	0.075^b^	43.841 ± 8.35	40.750 ± 7.27	0.254^a^
PCS^c^	49.851 ± 5.99	46.600 ± 4.67	0.007^a^	51.511 ± 5.52	47.269 ± 4.061	0.002^b^	48.163 ± 5.99	45.324 ± 5.71	0.301^a^
MCS^c^	44.665 ± 9.38	39.55 ± 8.97	0.010^a^	45.128 ± 9.70	39.510 ± 10.32	0.027^b^	44.194 ± 9.09	39.653 ± 6.05	0.116^b^

*Data presented in mean ± SD; >p75, symptoms of MSD in more than six regions in the last 12 months; QoL, quality of life; PF, physical functioning; RP, role limitations due to physical problems; BP, body pain; GH, general health perception; VT, vitality; SF, social functioning; RE, role limitations due to emotional problems; MH, mental health; PCS, physical component summary; MCS, mental component summary*.

a *Mann–Whitney test*.

b *t-test. p < 0.05*.

c *PCS and MCS score grouped on 50th percentile*.

Table 5 shows the association of the lowest scores in PCS and MCS (≤p50) with the highest prevalence MSD cases (>p75), %BF, and rural and urban areas, adjusted for age and gender. The participants with high MSD (p75) increased the risk of a reduced score on the MCS and PCS summary measurements (*p* < 0.05) for QoL. Also, rural teachers were associated with low PCS (*p* < 0.01). In addition, younger teachers (<45 years old), and female teachers are significantly associated with lower QoL perception in the MCS (*p* < 0.05) regardless area and obesity according to %BF.

**Table 5:** Logistic regression for the association between PCS and MCS of QoL with the 75th percentile of MSD, area, and body fat percentage adjusted for age and gender.

	**PCS (≤p50)**	***p***	**MCS (≤p50)**	***p***
	**OR [95% CI]**		**OR [95% CI]**	
>p75 MSD	4.859 [1.908 – 12.372]	0.001	2.931 [1.208 – 7.116]	0.017
Area (rural)	2.580 [1.218 - 5.465]	0.013	1.534 [0.727 – 3.237]	0.261
Gender (female)	1.691 [0.669 - 4.271]	0.266	2.622 [1.007 – 6.827]	0.048
Age (≤44years old)	0.680 [0.308 – 1.499]	0.340	2.289 [1.038 – 5.046]	0.040
%BF	0.996 [0.945 - 1.050]	0.895	0.951 [0.902 – 1.004]	0.074
Hosmer-Lemeshow	0.315		0.346	

*PCS, physical component summary; MCS, mental component summary; %BF, body fat percentage; OR, odds ratios [confidence interval]. p < 0.05*.

## Discussion

MSDs have been an important factor associated with teachers' mental and physical health. Thus, our objective was to determine the association of MSD with QoL considering sociodemographic and body composition factors in urban and rural Chilean teachers; 89% of teachers presented pain in some part of the body, close to the highest pain prevalence of 95% found in teachers worldwide according to Erick and Smith (35) and is slightly higher than that reported by Solis-Soto in urban and rural Bolivian teachers at 86%, as well as being drastically higher than the prevalence in the general Chilean working population at 49.8% and the nationwide rate reported in the Chilean National Health Survey [(42.6%, (36)]. QoL evaluation in this study showed that differences exist between teachers with higher and lower PCS scores according to their number of painful MSD areas, a factor associated with the QoL PCS reported in previous studies (11). MCS also presented differences according to painful areas. This mental QoL component was also affected in rural teachers when presenting from two simultaneous chronic health conditions (27); therefore, we observe a need to generate preventive measures for teacher health, as it affects both their mental and physical health.

Prior studies suggest that female gender is an MSD risk factor (36, 37). In this sense, female gender could be contribute to reporting MCS in the present study. On the other hand, rural female teachers also had a higher prevalence of housework at over 15 h per week. This is in addition to the overload outside of working hours and which can cause work–family conflicts in teachers (38), a relevant phenomenon that can lead to greater work burnout, stress, and mental disorders in people (39). These results are important, considering that in Chile this is a profession with a high proportion of women (27, 40, 41), so that preventive health protocols emphasizing female gender should exist.

In this study, MSD prevalence was similar in urban teachers (90.2%) compared with the rural teacher sample (87.3%), in contrast to Bolivia, where higher pain rates were reported in rural teachers [(86.4%; (20)]. However, one aspect to consider is that in Chile, most teachers work in urban areas (28), in contrast with what Solis-Soto reported in Bolivia.

Additionally, in this study, rural teachers reported high MSD rates in the neck (46.5%) and lower back (42.3%), both segments with high rates in teacher studies (35, 42). The hand/wrist segment also presented a 46.5% pain prevalence; this is especially high considering that they used the Nordic questionnaire like Bolivia with 29% in rural teachers (20), Malaysia with 9.9% in the total teacher sample (43); Turkey with 16% in the total sample (11), and Botswana with 30.7% (42). Hand/wrist pain in this study is the only one more prevalent in rural teachers than in urban teachers (46.5 and 43.9%, respectively), which might be related to rural school infrastructure, depression, or psychosocial factors (4, 19, 20). In this regard, further studies are needed to report on high carpal area problem prevalence. However, this high prevalence also indicates that prevention and safety measures for teachers are necessary to avoid disorders like carpal tunnel syndrome, since it is common for it to appear during working age due to comfort, gender, or repetitive movement exposure factors in the general population (44).

Regarding urban teachers, the results for lower back (70.7%), neck (64.6%), shoulders (51.2%), and neck/shoulders together (76.8%) showed the highest pain prevalence in this study. This was higher than the rates reported by teachers in China with 48.7% in the neck and 45.6% in the neck/shoulders (23); Turkey with 43.8% in the lower back, 42.5% in the neck, and 43.8% in the shoulders (45); and Brazil with 49.6% in the lower back and 50.2% in the neck (19). These results place Chilean urban teachers among those with the highest MSD prevalence in these body segments, similar to teachers in Nigeria with 62.3% in the shoulders and 57.9% in the neck (12); Saudi Arabia with 63.8% in the lower back, 45.4% in the shoulders, and 42.1% in the neck (16); or Kenya with 64.98% in the lower back (21). It should be considered that in this study all urban teachers did classes in primary and/or secondary schools. By contrast, the rural teachers only taught primary school, which could explain the high pain rates associated with PCS. In this regard, secondary teachers are reported to present greater MSD risk factors (14, 37), which may be due to work overload, stress, and behavior specific to the adolescent development of secondary students.

In this study, overweight and obesity rates are high without distinction for gender or for higher/lower QoL scores. This may explain the high prevalence of knee pain (urban: 41.5%; rural: 40.8%) and lower back pain (urban: 70.7%; rural: 42.3%) in the total sample. These symptoms were associated with being overweight or obese in prior studies (14, 43). In this sense, high MSD prevalence in the lower back and knees could be due to the overload teachers present due to the high prevalence of obesity reported. Additionally, it has been observed that teachers who do not perform physical activity or sport present more significant obesity than those who do perform physical activity or sport (46). This observation of low physical activity or sport in teachers could also contribute to an increase in MSD due to a low strengthening of the muscular system. BMI also exposes an underestimation in obesity prevalence faced with %BF (47); in this case, BMI presents half of the obese in the face of %BF (16.8% and 45.4% respectively) among teachers with low scores (≤p50) in the physical component. In this sense, various studies worked with BMI in association with specific pain regions, such as lower back or knee (15, 43). However, evaluating obesity by %BF allows us to observe a higher rate, since BMI does not consider all human body components (47). In future studies, we suggest evaluating body component evaluation given this BMI underestimation.

QoL perception in rural teachers presents no differences according to the evaluations between those who suffer a number of painful areas ≤p75 and >p75, but it can be observed that the most severe MSD cases (>p75) have low evaluations in all QoL summary scales and measures. Among urban teachers, differences arise in the MCS and PCS measurements among those with an MSD number ≤p75 and >p75, with all QoL scales affected except for MH and VT. This suggests work overload differences between urban and rural teachers. The QoL PCS shows a strong association with the presence of over six painful areas (>p75) and the urban area. MCS shows only a significant association with the group with more MSD as well as with younger teachers, and female gender. This background is consistent with the literature that indicates MSD as a risk factor for suffering mental disorders, psychological stress, and diminished biopsychosocial QoL among teachers, especially on female teachers (4, 10, 17). Besides, these data demonstrate the differences in working conditions between urban and rural teachers and the diversity of activities they perform that can affect their physical and mental health. Urban teachers with high prevalence of MSD may be more affected than rural teachers because they may be subjected to more stressful working conditions such as larger numbers of students, problems in educational resources, and working in more complex organizations that may affect the work climate (48, 49). However, rural teachers report more risk to have a lower QoL PCS perception. On the other hand, it is interesting that younger teachers present more significant mental health problems than older teachers. Younger teachers may present this due to a low level of stressor management and/or self-perception with lower capacities to lead a class. In contrast, older teachers may have developed tools to better cope with stressors through teaching experience. However, these results are of particular concern since it has been documented that teachers in the first years of teaching work are the most vulnerable to leaving the profession (50, 51). Therefore, it has been shown that work stress in public schools affects teachers' physical and mental health, mainly teachers in urban areas and younger individuals. Therefore, it is relevant to generate interventions based on physical and mental aspects and to provide adequate tools to manage stress during initial teacher training.

### Limitations

This study has various limitations. The first is typical of cross-sectional studies, meaning that in the future longitudinal studies will be needed to follow up on the variables that influence teacher MSD. Secondly, the QoL and MSD questionnaires used were based on self-reporting, impeding derivation of any causality from reported associations. However, we had a high teacher reply rate. These instruments are also widely used by researchers and validated in the country of application (32, 34). Thirdly, we studied a representative sample of rural teachers but only worked in three rural areas of one Chilean region, although one rural area was added according to studies prior to our group (27, 46). This may limit generalization of study results to other regions. However, in the following study, we included urban teachers, allowing comparison between the two areas. Although important MSD studies exist for urban teachers (19, 43, 52, 53), few studies compare urban and rural teachers (20); thus, the present study has the strength of being a pioneering study involving urban and rural teachers, MSD, QoL, and body composition.

## Conclusion

Urban and rural teachers present high rates of MSD and obesity. High MSD rates affect the physical and mental components of QoL. This suggests that working conditions are negatively impacting the health of urban and rural teachers. We suggest considering the results of this study to generate preventive and teacher health monitoring measures to protect their QoL and properly undertake their labors.

## Data Availability Statement

The raw data supporting the conclusions of this article will be made available by the authors, without undue reservation.

## Ethics Statement

The studies involving human participants were reviewed and approved by Bioethics committee at Pontificia Universidad Católica de Valparaíso. The patients/participants provided their written informed consent to participate in this study.

## Author Contributions

PL designed the study. PL, PC, and GV-F performed the measurements, processed the data, drafted the manuscript, and designed the tables. GV-F, PL, LL, and BL performed the statistical analysis. All authors discussed the results and commented on the manuscript.

## Conflict of Interest

The authors declare that the research was conducted in the absence of any commercial or financial relationships that could be construed as a potential conflict of interest.
